# Recombinant Expression and Characterization of a Novel Type I Baeyer–Villiger Monooxygenase from a *Streptomyces* Strain Isolated from the Rhizosphere of the Atacama Desert *Lupinus oreophilus*

**DOI:** 10.3390/ijms26135940

**Published:** 2025-06-20

**Authors:** Carolina González, Sebastián Rodríguez, José Pablo Reyes-Godoy, Valeria Razmilic, Irene Martínez

**Affiliations:** Centre for Biotechnology and Bioengineering (CeBiB), Department of Chemical Engineering, Biotechnology and Materials, University of Chile, Santiago 8370459, Chile; carolina.gonzalez.al@ug.uchile.cl (C.G.); sebastian.rodriguez@cebib.cl (S.R.); joreyes@ug.uchile.cl (J.P.R.-G.);

**Keywords:** Baeyer–Villiger monooxygenase, *Streptomyces*, Atacama Desert, *E. coli* Shuffle T7, heterologous expression

## Abstract

The Atacama Desert is emerging as an unexpected source of microbial life and, thus, a source of bioactive compounds and novel enzymes. Baeyer–Villiger monooxygenases (BVMOs), a subclass of flavin-dependent monooxygenases (FPMOs), have gained attention as promising biocatalysts for the biosynthesis of industrially relevant molecules for a wide range of applications, such as pharmaceuticals and polymers, among others. BVMOs catalyze the oxidation of ketones and cyclic ketones to esters and lactones, respectively, by using molecular oxygen and NAD(P)H. BVMOs may also catalyze heteroatoms oxidation including sulfoxidations and *N*-oxidations. This work aims to search for novel BVMOs in the genomes of new bacterial strains isolated from the Atacama Desert. Bioinformatic analysis led to the identification of 10 putative BVMOs, where the monooxygenase named MO-G35A was selected. Genome context showed, downstream of the MO-G35A, a gene encoding for an enzyme from the short-chain dehydrogenase/reductase family, suggesting a closer redox loop between both enzymes. MO-G35A was successfully expressed in three *Escherichia coli* expression systems, where higher yields were achieved using the *E. coli* Shuffle T7 as host, suggesting that correct disulfide bond formation is necessary for correct folding. Enzyme characterization showed that it operates optimally at 35–38 °C, exhibiting a *Km* of 0.06 mM and a *kcat* of 0.15 s^−1^ for bicyclo [3.2.0] hept-2-en-6-one (BHC). Furthermore, the study revealed high stability in the presence of organic solvents, making it suitable for applications in various industrial processes, especially when the substrates have poor solubility in aqueous solutions. These results highlight the robustness and adaptability of enzymes in extreme environments, making them valuable candidates for biotechnological applications.

## 1. Introduction

Flavin-dependent monooxygenases (FPMOs) are involved in a wide range of biological processes. Many of these enzymes play a key role in the catabolism of natural and anthropogenic compounds; they also participate in the biosynthesis of hormones, vitamins, antibiotics, and other natural products [1,2]. The classification of FPMOs is based on structural features, protein sequence motifs, electron donor, and type of oxygenation reaction [3,4,5].

The present work focuses on a special group of these enzymes called Group B FPMOs, and particularly on the Baeyer–Villiger Monooxygenases (BVMOs) that belong to this group. BVMOs are usually present in bacteria and fungi [6], participating in a reaction that inserts an oxygen atom into C-C bonds adjacent to a carbonyl group, generating esters or lactones [7]. Extensive research on group B BVMOs has shown that they carry out oxidations on a wide variety of substrates with exquisite chemo-, regio-, and enantioselectivity [8,9,10].

BVMOs have gained attention as promising biocatalysts for the biotechnology and pharmaceutical industries, as they can be applied to a diverse set of reactions, such as the conversion of a wide range of ketones into esters or lactones and for the synthesis of fatty acids [11], biopolymer precursors [12], synthesis of aroma compounds [13] and others [14]. These include sulfoxidation reactions, to produce optically active sulfoxides such as Esomeprazole, a triazole drug used in the treatment of gastroesophageal reflux disorders [15,16], and in the oxygenation of heteroatoms for the biosynthesis of drug metabolites [17,18]; for example, to produce molecules with the characteristic bicyclo [3.2.0] ring structure [19].

Additionally, over the past decade, extremophiles have gained interest in the search for novel enzymes [20], including BVMOs [21]. Evolution may have selected enzymes that can perform in these harsh environmental conditions, which could help solve some industry challenges related to low enzyme stability [20] and low solvent tolerance [21]. Thus, different BVMOs have been isolated from different extremophiles, like *Thermobifida fusca* [22], *Thermocrispum municipale* [23], and more recently, *Streptomyces leeuwenhoekii* [21] isolated from the Atacama Desert.

The Atacama Desert is an extreme environment and the driest desert on Earth [24], located in the northern region of Chile, where different extreme habitats are present, such as salars and geysers, among others. Its core region was described as featuring Mars-like soils, deemed too extreme for life to exist due to extreme aridity, high levels of UV radiation, the presence of inorganic oxidants, high salinity, and very low concentrations of organic carbon [25]. However, subsequent investigations, made in the same hyper-arid core, confirmed that several taxa of cultivable microorganisms, especially Actinobacteria, live in this harsh environment [26,27]. Moreover, some of these Actinobacteria produce novel bioactive secondary metabolites, such as Chaxamycins [28] and Asenjonamide [29], with confirmed antibacterial activity. These findings show that the Atacama Desert is an unexpectedly rich resource of Actinobacterial diversity and novel chemicals [30].

Recently, research groups isolated new Actinobacteria, six *Streptomyces* and four *Micromonospora* from the Atacama Desert *Lupinus oreophilus* rhizome. It is well reported that the rhizome-associated Actinobacteria can directly promote plant growth by producing different phytohormones such as indole-3-acetic acid, cytokinins, and gibberellins [31]. Due to the relation between this phylum and the production of natural compounds, their genomes were sequenced for metabolic pathway mining in search of new antibiotics or bioactive compounds [6,32]. Considering the availability of these genomes, their provenance, and the relevance of BVMOs in the biosynthetic pathways of natural compounds, it was decided to perform genome mining to find and express novel BVMOs from this extreme environment.

This work is focused on the bioinformatic search and selection of a novel putative BVMO from Actinobacteria isolated from the Atacama Desert, its comparative heterologous expression in different *Escherichia coli* expression systems, and its biochemical characterization. Bioinformatic analysis led to the identification of 10 putative BVMOs. The putative enzyme denominated MO-G35A from *Streptomyces* sp. *G3A*, which showed significant similarity to known BVMOs, was selected. Phylogenetic analysis revealed that MO-G35A is closely related to Sle_62070 from *Streptomyces leeuwenhoekii*, which is known for its high activity on phenylacetone and bicyclo [3.2.0] hept-2-en-6-one (BHC). For recombinant protein production, including BVMOs, *E. coli* is the most widely preferred host organism [33]. However, the heterologous expression of novel enzymes in *E. coli* can be hindered by several factors, such as insoluble expression, low yields, lack of posttranslational modifications, codon usage bias, etc. To overcome some of these limitations, three different *E. coli*-based expression systems with codon-optimized genes were tested and compared for the expression of the novel BVMO found in an Actinobacterial strain isolated from the lupine rhizosphere. These expression systems considered the expression of the target enzyme in the periplasm, cytoplasm, or in an oxidant cytoplasm environment, the latter provided by the *E. coli* Shuffle T7 strain. The novel BVMO was successfully produced, and its properties were characterized using NADPH and BHC, unveiling its catalytic potential. The results emphasize the robustness and adaptability of enzymes in extreme environments, turning them into valuable candidates for novel biotechnological applications.

## 2. Results and Discussion

### 2.1. Identification of a Type I BVMO Using Computational Analysis

A bioinformatic search for putative type I BVMOs was conducted using 10 genomic sequences obtained from six *Micromonosporas* and four *Streptomyces* strains isolated from the *Lupinus oreophilus* rhizosphere at the Atacama Desert, Chile [34]. The query sequences were based on type I BVMO sequences described in Gran-Scheuch et al. (2018) [21] and Romero et al. (2016) [23]. From the search, 10 putative type I BVMOs sequences were selected, all exhibiting an identity percentage greater than 35% and an E-value close to 0. These sequences were analyzed using the InterPro database and diagnostic tool (https://www.ebi.ac.uk/interpro/, accessed on 1 March 2024). The analysis revealed conserved domains in these enzymes and their functional characteristics, leading to the selection of a putative type I BVMO enzyme for further bioinformatics analysis, expression, and characterization. The selected type I BVMO putative enzyme from *Streptomyces* sp. *G35A* (MO-G35A) had a 48% and 86% identity with the query amino acid sequences Sle_13190 and Sle_62070 (reported in Gran-Scheuch et al. (2018) [21]), respectively. (The Whole Genome Shotgun project is available at DDBJ/ENA/GenBank under the accession JBNBZQ000000000. The version used in this paper is version JBNBZQ010000000.)

**Figure 1:**
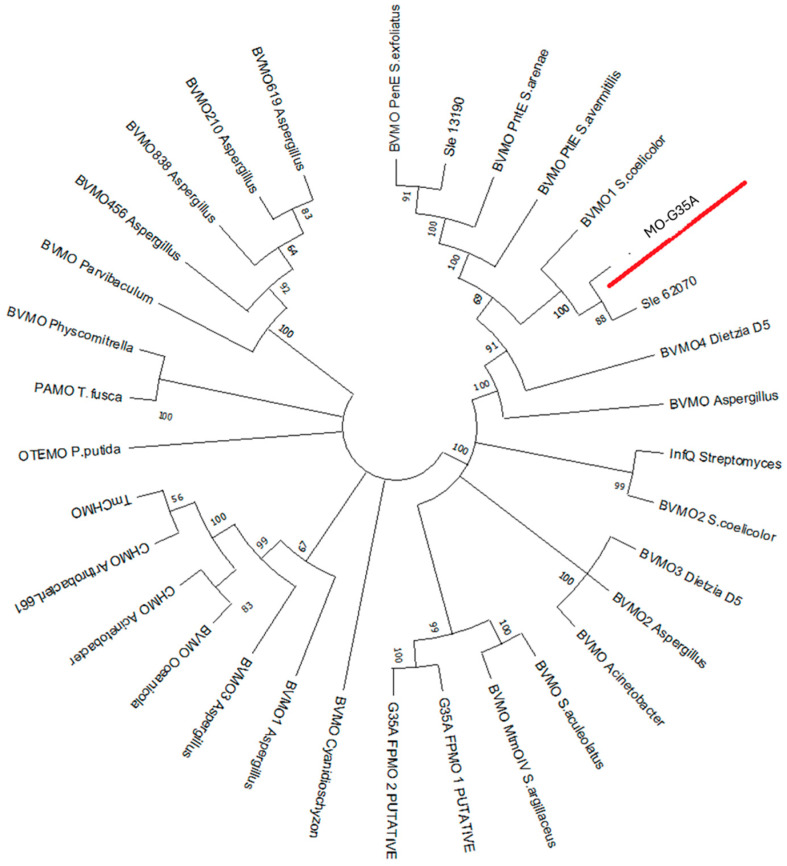
Phylogenetic analysis of MO-G35A (underlined in red) with other type I BVMOs identified and characterized in previous research. These amino acid sequences were extracted from NCBI with the accession codes reported in the supplementary material of Yamamoto et al. (2021) [35]. The construction of the phylogenetic tree was initiated with a ClustalW alignment of all amino acid sequences. The neighbor-joining method [37] was implemented in MEGA-X software Version 11.0.13, and the bootstrap consensus tree was inferred from 1000 replicates [38].

A phylogenetic tree was constructed using the neighbor-joining method in MEGA-X software Version 11.0.13, with the amino acid sequence of MO-G35A along with several type I BVMOs identified and characterized in previous research. These amino acid sequences were extracted from NCBI with the accession codes reported in the supplementary material of Yamamoto et al. (2021) [35]. The construction illustrated in Figure 1 indicates that the phylogenetic position of MO-G35A is most closely related to the BVMO query sequence Sle_62070 from *Streptomyces leeuwenhoekii*, sharing an 86% amino acid identity. The corresponding enzyme has shown to have a high activity for phenylacetone and BHC [21], and a close relationship was also observed with the enzyme BVMO1 from *Streptomyces coelicolor* that showed sulfoxidation activity and oxidation activity for thioanisole and BHC, using whole cell biocatalysis [36].

**Figure 2:**
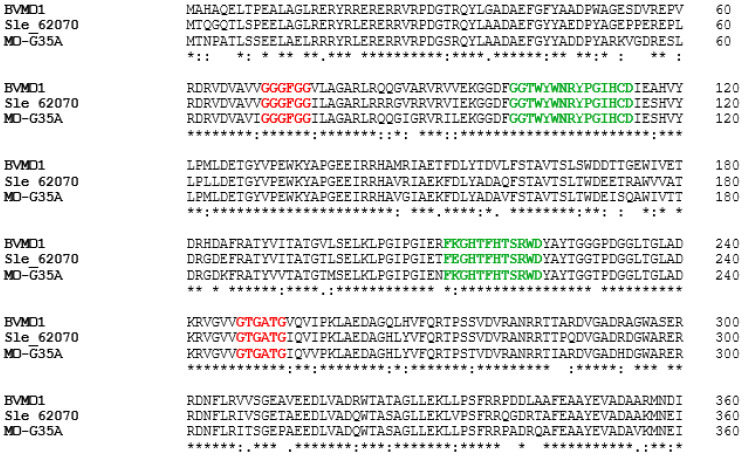
Partial alignment of the conserved domain region of MO-G35A and two type I BVMOs previously described, Sle_62070 from *Streptomyces leeuwenhoekii* [21] and BVMO1 from *Streptomyces coelicolor* [36]. The Rossman domains are shown in red, and the typical BVMO is shown in green.

Based on the phylogenetic tree results, the two closest enzymes were selected to make an alignment with MO-G35A in InterPro (Sle_62070 from *Streptomyces leeuwenhoekii* and BVMO1 from *Streptomyces coelicolor*), to check the presence of typically conserved domains. In Figure 2, in red, two conserved Rossmann sequence motifs (GXGXX[G/A]) are observed, where the first one is close to the N-terminal end and the second one is in the middle of the amino acid sequence, clearly distinguishing these enzymes from the mechanistically related flavoprotein hydroxylases [39]. Two conserved sequences of type I BVMOs have also been identified and are shown in green in Figure 2; the sequence (FXGXXXHXXXW[P/D]) allows differentiation from the other subclass, called flavin-containing monooxygenases (FMOs), whose conserved sequence is similar (FXGXXXHXXX[Y/F]), differing by only one amino acid [40]. Also, the conserved sequence ([A/G]GxWxxxx[F/Y]P[G/M]xxxD), located between the N-terminal Rossman domain and the aforementioned type I BVMO sequence, has been reported to be part of the active site of BVMOs [41,42].

**Figure 3:**
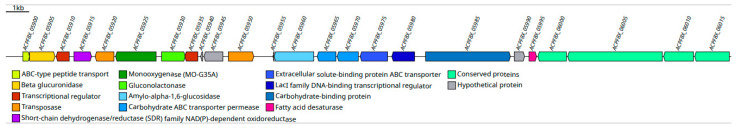
Protein-coding sequences of the 31 kb contig of *Streptomyces* sp. G35A, which contains the *MO-G35A (ACPF8X_05925) gene*. ACPF8X_05905 contains the domain of ABC transporter and a domain of beta-glucuronidase.

It was determined that MO-G35A is located at the beginning of an approximately 31 kb contig (Figure 3). Analysis of the surrounding protein-coding sequences showed transcriptional regulators and transposon elements located upstream and downstream of the *MO-G35A gene*. Specifically, upstream of the monooxygenase gene, the contig has genes associated with the degradation of glycogen (blueish genes). Downstream of the *MO-G35A gene*, there is a peptide ABC transporter, a beta-glucuronidase, and a gene encoding for the short-chain dehydrogenase/reductase (SDR) family NAD(P)-dependent oxidoreductase. The latter is relevant since the function of this enzyme requires NAD(P)+ cofactor, which is produced during the action of MO-G35A oxidizing NADPH. Thus, the catalysis of both reactions could help maintain the redox balance. The fusion of monooxygenases with dehydrogenases has been reported in other studies to create independent redox cascade reactions [43,44,45].

The protein-coding sequences of the 31 kb contig of *Streptomyces* sp. G35A, which contains the MO-G35A gene *G35Ag_06724, contains a domain of ABC transporter and a domain of beta-glucuronidase.

Therefore, even though it is not possible to determine the substrate of MO-G35A in the bacterial metabolism given the lack of genetic information downstream of the gene, it is proposed that the SDR NADP-dependent oxidoreductase could be functioning coupled to the enzymatic MO-G35A activity.

### 2.2. MO-G35A Expression Systems Comparison

For the expression of the selected type I BVMO MO-G35A, three different expression systems based on *E. coli* were tested. For the expression in the periplasm, the MO-G35A gene was cloned in frame with the pelB signal sequence of the pET-22b plasmid and used to transform the *E. coli* NiCo21(DE3) strain. This strain is derived from *E. coli* BL21(DE3) and has been engineered to minimize protein contamination when purified by immobilized metal affinity chromatography [46] and has been claimed to be a superior alternative to *E. coli* BL21(DE3) for routine protein expression by the provider (New England Biolabs).

For the expression in the cytoplasm of *E. coli*, the MO-G35A gene was cloned into the pET-28a plasmid and used to transform *E. coli* NiCo21(DE3), for the expression in the canonical reductive cytoplasm environment, and the same construct was transformed into *E. coli* Shuffle T7 for the expression in an oxidative cytoplasm. The last strain contains knockouts in the genes *trxB* and *gor*, this double mutant is non-viable; however, its viability is restored by introducing the AphlC* protein that uses NADH instead of NADPH, reestablishing the generation of NAD+ [47]. Thus, these modifications result in the decoupling of the cytosolic pool of NADPH and the disulfide bond reductases activity [48], producing an oxidative environment capable of forming disulfide bonds in the cytoplasm of *E. coli*. Furthermore, *E. coli* Shuffle T7 strains express the DsbC into the cytoplasm, a chaperone disulfide bond isomerase natively found in the periplasm of *E. coli*, that can assist with the folding and the rearrangement of mislocated disulfide bonds [47].

**Figure 4:**
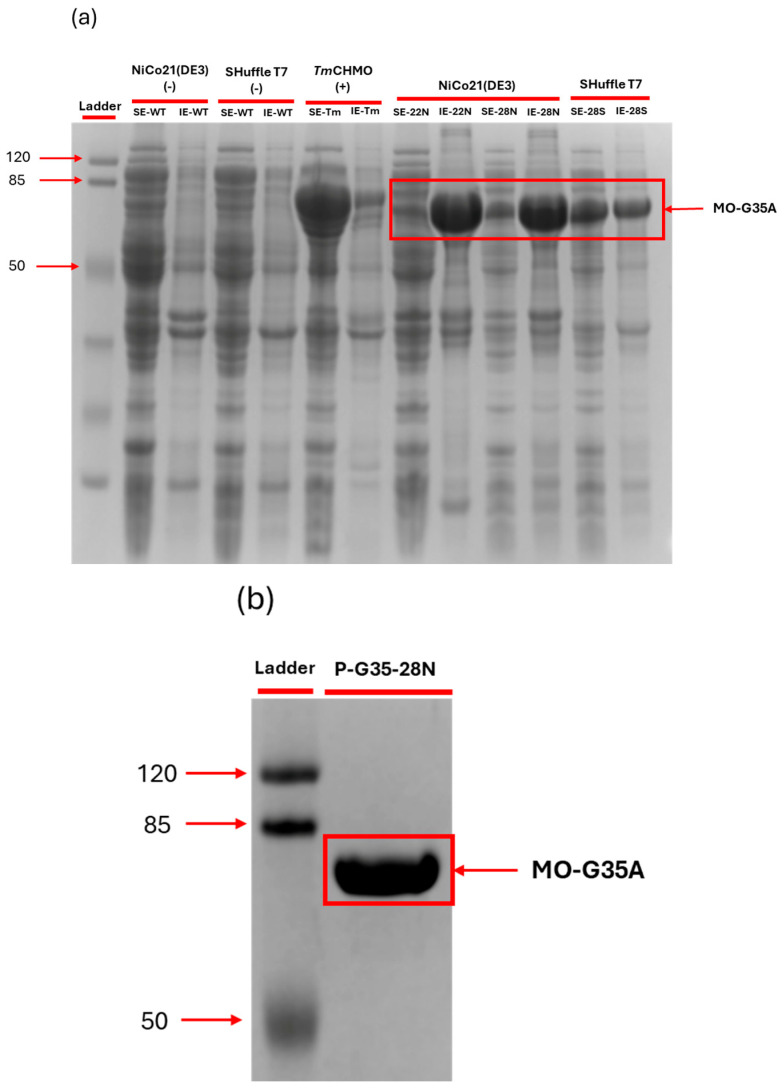
SDS-PAGE: (**a**) Analysis of MO-G35A produced in each expression system, with negative and positive controls. The lane titles indicate: Ladder: Protein Molecular Weight Ladder; for each host, WT indicates the wild-type (host) strain, SE is the soluble extract, and IE is the insoluble extract of each strain; 22 N and 28 N references *E. coli* NiCo21(DE3) expressing MO-G35A with pET-22b and pET28a plasmids, respectively, while 28S indicates the *E. coli* Shuffle T7 strain transformed with pET28a expressing MO-G35A; Tm indicates the positive control for soluble expression of *Termocrispum municipale* CHMO expressed in *E. coli* Top10. The MO-G35A band is inside the red box at about 73 kDa. (**b**) Purified MO-G35A.

MO-G35A was successfully cloned and produced by both *E. coli* host strains (NiCo21(DE3) and Shuffle T7). Figure 4 shows the SDS-PAGE with the 73 kDa band corresponding to MO-G35A, matching the theoretical molecular weight of the protein. A significant amount of MO-G35A is produced in the insoluble fraction for all expression systems; this is commonly reported when a strong promoter, such as T7 promoter, is used to command the expression of the recombinant protein [49]. The band’s intensity in the insoluble fraction is notorious for cases in which the *E. coli* NiCo21(DE3) host strain is used for the expression in the periplasm or in its reductive cytoplasm. However, a slight band can be observed in the soluble fractions for this strain. On the other hand, when the *E. coli* Shuffle T7 host strain was used, an important band could be observed in the soluble fraction, making it a promising strain for whole-cell biocatalysis.

**Figure 5:**
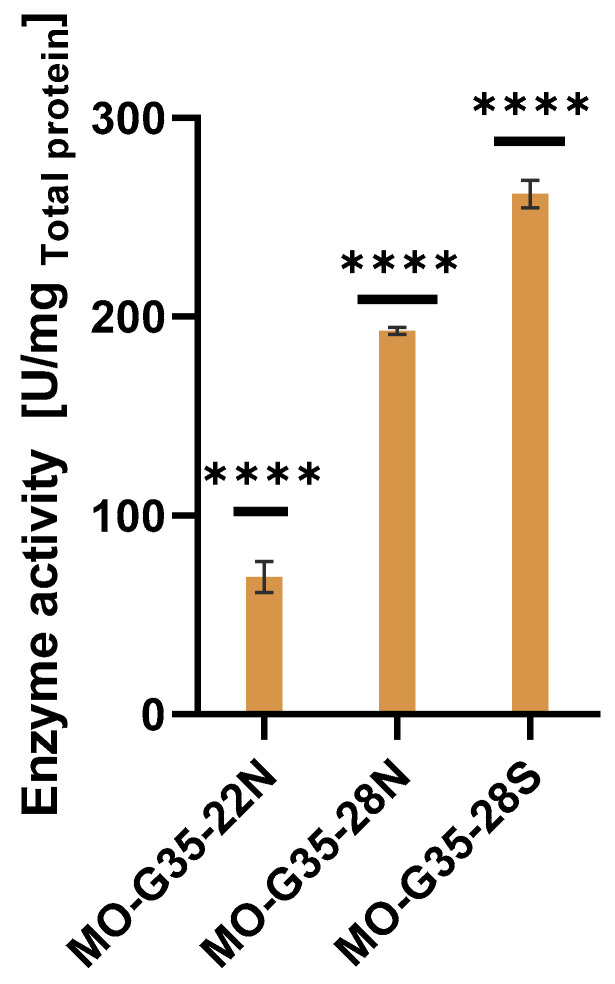
Crude extracts’ enzymatic activity for MO-G35A produced by each expression system. The enzyme names refer to the expression system used; i.e., 22 indicates that the plasmid used was the pET-22b, while 28 refers to pET28a. The final letter indicates the corresponding host: N for *E. coli* NiCo21(DE3*)* and S for *E. coli* Shuffle T7. Enzymatic activity for MO-G35A produced in each expression system was compared using one-way ANOVA followed by a Tukey’s multiple comparisons test, considering *p* < 0.05 for significant differences. Four asterisks represent a statistically significant difference if *p* < 0.0001.

The enzyme activity was determined using crude extracts for all expression systems using BHC as substrate (Figure 5). The highest specific enzyme activity was attained with the *E. coli* Shuffle T7 host strain, where its activity was 32% and 282% higher than the activity shown when *E. coli* NiCo21(DE3) was used with cytoplasmic and periplasmic expression, respectively. This result suggests that MO-G35A contains disulfide bridges in its structure and that *E. coli* Shuffle T7 oxidant cytoplasm favors the correct folding of the enzyme, incorporating the proper disulfide bridges. To corroborate the presence of disulfide bridges in the structure of MO-G35A, disulfide bridges were predicted using Disulfide by Design 2.0 [50], where one disulfide bridge was predicted between CYS 285 and CYS 552. Moreover, the region where these cysteines are located is a region with a high calculated B-factor value, which means that this disulfide bridge could be involved in the stability of the enzyme and thus its proper folding. Another advantage of the *E. coli* Shuffle T7 strain is the expression of the intracellular DsbC, which works as a disulfide bond isomerase, but also can act as a chaperone, helping with protein folding in general [51].

### 2.3. MO-G35A Biochemical Characterization

**Figure 6:**
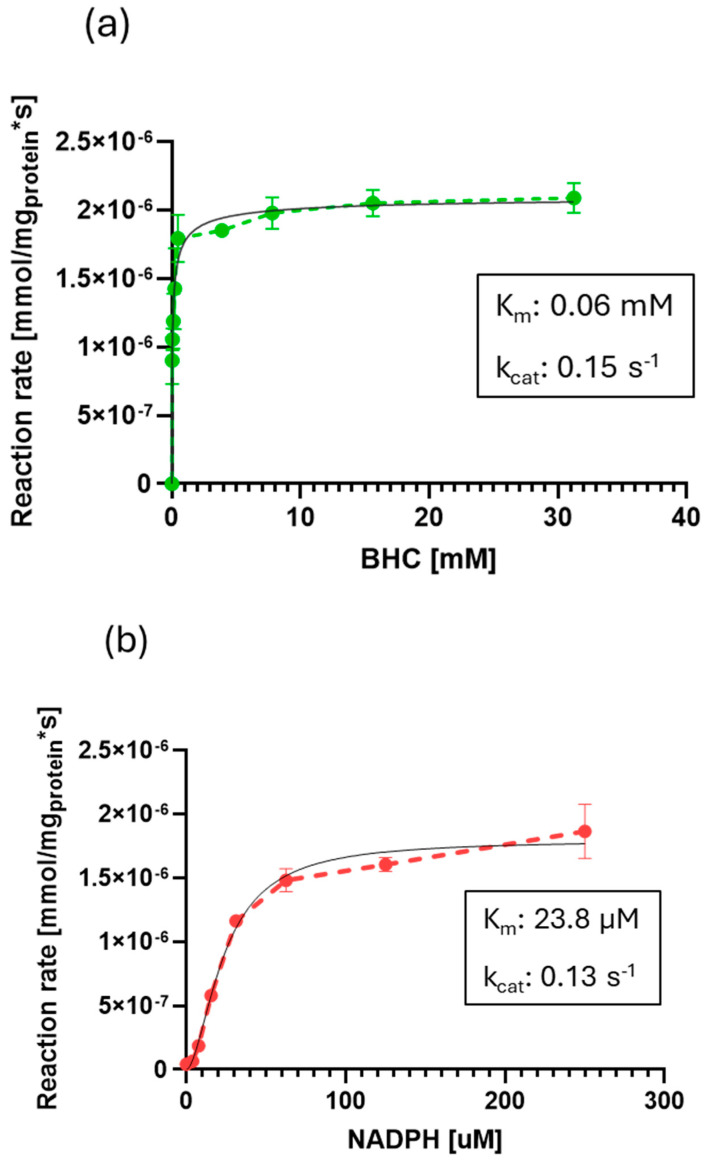
MO-G35A enzymatic reaction rate at different (**a**) BHC (bicyclo [3.2.0] hept-2-en-6-one) or (**b**) NADPH initial concentrations. The continuous lines indicate the sigmoidal adjustment for kinetic parameters determination.

The MO-G35A was purified and used to perform activity assays for the enzyme biochemical characterization. Figure 6 shows the enzymatic reaction rate with varied BHC and NADPH concentrations in the reaction mixture, at pH 7.5 and 35 °C. The kinetic parameters were calculated using the standard Michaelis–Menten method (R^2^_NADPH_ = 0.96; R^2^_BHC_ = 0.93) and an allosteric sigmoidal adjustment (R^2^_NADPH_ = 0.98; R^2^_BHC_ = 0.95). The following reported parameters come from the latter, as the best correlation factors were obtained with said model for both substrates. Thus, the parameters for BHC were a *Km* of 0.06 mM, a *kcat* of 0.15 s^−1^ and a catalytic efficiency (*kcat*/*Km*) of 2.5 mM^−1^ s^−1^. Meanwhile, for NADPH the *Km* value was 23.8 μM, the *kcat* 0.13 s^−1^ and the catalytic efficiency 5.46 × 10^−3^ μM^−1^ s^−1^.

Previously, Gran-Scheuch et al. (2018) [21] had reported the kinetic parameters for one of the proteins used as a query sequence (Sle_62070) for MO-G35A. With BHC as substrate, the *Km* reported (0.2 mM) was greater than the one obtained for MO-G35A, which indicates a higher affinity of MO-G35 for BHC. However, MO-G35A had a 26-fold lower *kcat* than Sle_62070, resulting in an 8-fold lower catalytic efficiency. On the other hand, Jensen et al. (2012) [52] reported values for kinetic parameters considering NADPH and BHC as variable and fixed substrate concentration, respectively, for a flavin-containing monooxygenase from *Stenotrophomonas maltophilia* (SMFMO). The reported values were 27.3 μM for *Km* and 0.022 s^−1^ for *kcat* [52], which shows that MO-G35A has 6-fold higher catalytic efficiency.

**Figure 7:**
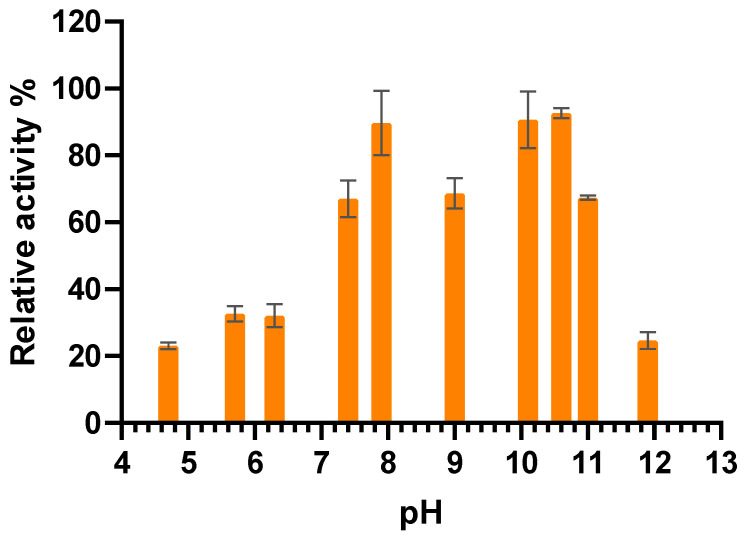
The pH effect on MO-G35A activity.

The pH effect on MO-G35A activity was determined at 35 °C (Figure 7). The enzyme has its highest activity in the pH range 7.5 to 10.6, while its activity decreases significantly outside the mentioned pH range. This performance is highly related to that of other characterized BVMOs [21,50,51].

**Figure 8:**
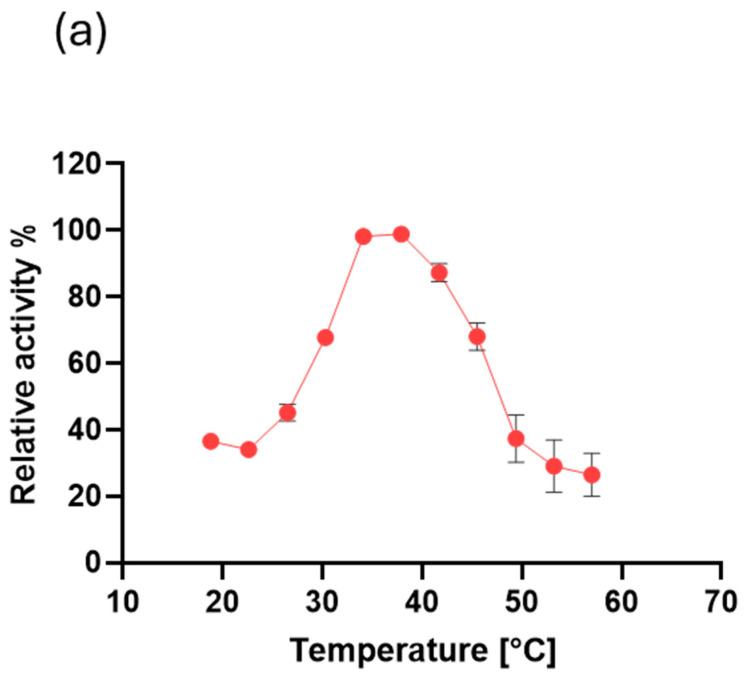

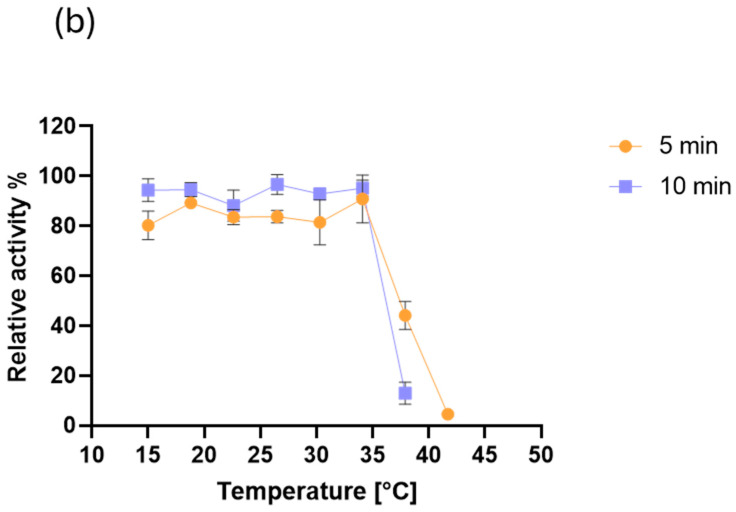
Influence of temperature on MO-G35A activity and MO-G35A thermal stability. (**a**) Effect of temperature on MO-G35A activity. The activity assay was performed using BHC as substrate, and relative activity was calculated using 100% as the highest registered activity (obtained at 38 °C). (**b**) Thermal stability of MO-G35A. The purified enzyme was incubated for 5 and 10 min at different temperatures, and then the activity assay was performed at 35 °C. Relative activity was calculated using the measured activity at 30 °C as 100%.

Figure 8a shows how temperature affects MO-G35A performance in an activity assay using BHC as a substrate, at pH 7.5. The temperature range between 30 and 45 °C showed enzyme activity higher than 50% of the maximum, where the maximum was reached at 35–38 °C. The enzyme activity at temperatures outside the mentioned range decreases significantly.

Thermal stability results are shown in Figure 8b, where activity assays were performed at 35 °C and pH 7.5 after the enzyme had been heated at different temperatures for 5 or 10 min. It is shown that at temperatures between 15 and 35 °C, MO-G35A retains more than 80% of its activity, given any of the tested heating times. However, over 35 °C, remaining activity dramatically drops, especially when a 10 min heating time is used.

The optimal temperature range of MO-G35A is within the typical ranges reported for various BVMOs [53,54,55,56]. Moreover, it does not demonstrate a thermostable profile or optimal activity at high temperature values as those reported for more thermophilic counterparts [22,23]. However, its stability at 15–35 °C temperatures represents an advantage for its use in bioprocesses at ambient temperatures. This is especially important when reactants or products are thermally sensitive, to avoid unwanted side reactions, or to avoid the energy costs associated with rising temperatures [57].

**Figure 9:**
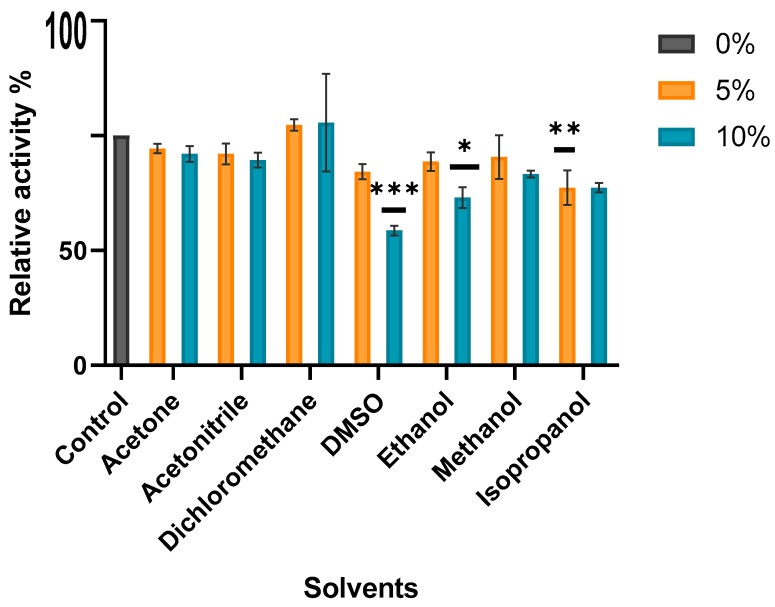
Effect of different solvents at 5 and 10% v/v concentration on recombinant MO-G35A activity. Relative activity was calculated using the no-solvent-containing assay as 100%. All solvents at 5% concentration were compared with the control by one-way ANOVA followed by a Tukey’s multiple comparisons test when significant differences (*p* < 0.05) of means occurred. The same analysis was performed on all solvents at 10% concentration. One, two, and three asterisks represent differences statistically significant for *p* < 0.05, *p* < 0.01, and *p* < 0.001, respectively.

Further enzyme characterization was performed by analyzing the effect of several water-miscible organic solvents on MO-G35A enzymatic activity (Figure 9). The enzyme proved to have a high tolerance to 5 and 10% v/v of acetone, acetonitrile, dichloromethane, and methanol, where the relative activity was 80–100% of the control. Meanwhile, DMSO caused the most significant inhibition among the tested solvents, especially at 10% v/v concentration, where about 40% reduction in enzyme activity was observed. MO-G35A showed tolerance to ethanol and acetonitrile, like the robust *Thermobifida fusca* phenylacetone monooxygenase (*tf*PAMO) [58] and retained high activity at 10% v/v. BVMOs have great potential for developing more sustainable industrial synthesis. However, the low solubility of substrates in aqueous media represents an industrial challenge, as it limits the catalytic efficiency of biocatalysts. Using cosolvents offers a solution by improving the solubility of hydrophobic compounds, increasing the reaction yield. Therefore, enzymes with high stability in the presence of organic solvents are highly valued in these industrial applications [18,59].

**Figure 10:**
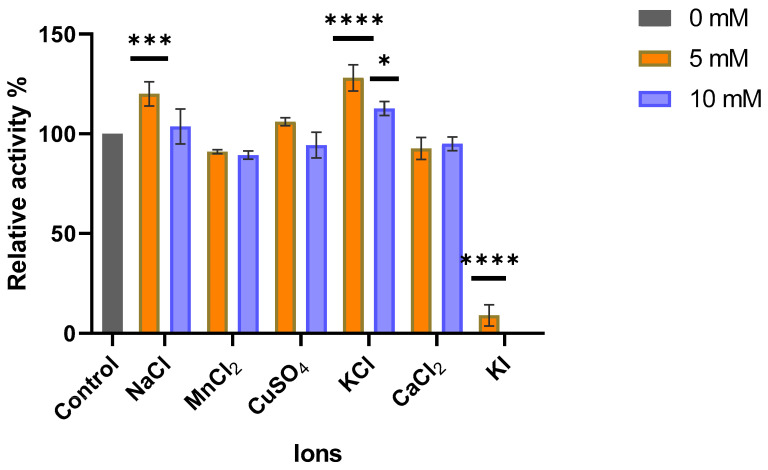
Effect of different salts at 5 and 10 mM concentrations on recombinant MO-G35A activity. Relative activity was calculated using enzyme activity with no salt added as 100% (control). All salts at 5 mM concentration were compared with the control by one-way ANOVA followed by a Tukey’s multiple comparisons test when significant differences (*p* < 0.05) of means occurred. The same methodology was followed for experiments with each salt at 10 mM. One, three, and four asterisks represent differences statistically significant for *p* < 0.05, *p* < 0.001, and *p* < 0.0001, respectively.

Figure 10 shows how NaCl and KCl salts enhance MO-G35A enzymatic activity, especially at a 5 mM concentration. Meanwhile, the figure also indicates that KI inactivates the enzyme at both tested concentrations. Despite the latter, MO-G35A showed, in general, high tolerance at the salts concentrations used in this study, this contrasts with the previously reported behavior for MO-G35A homologous enzymes, such as *Rhodococcus aetherivorans* Baeyer–Villiger monooxygenase (*Ra*BVMO) and *Amycolatopsis methanolica* Baeyer–Villiger monooxygenase (*Am*BVMO), which showed inactivation in the presence of copper, manganese, and potassium [60].

## 3. Materials and Methods

### 3.1. Chemicals or Reagents

Bradford reagent (protein assay dye) was purchased from Bio-Rad (Hercules, CA, USA). All other reagents used were purchased from Merck (Darmstadt, Germany).

### 3.2. Bioinformatic Search for Putative Type I BVMOs

Sequenced genomes, from strains isolated from the *Lupinus oreophilus* rhizosphere in the Atacama Desert, were obtained using the methodology described in Astakala et al. (2022) [34]. A local bioinformatic search for putative FPMOs was performed, using the genomic sequences of 10 isolated strains: four *Streptomyces* and six *Micromonospora*. The BLAST software Version 2.12.0 was used, and the query sequences were protein-coding sequences of BVMOs from *Streptomyces leeuwenhoekii* C34 (“Sle_13190”, “Sle_62070”) and a cyclohexanone monooxygenase from *Thermocrispum municipale* (WP_028849141.1). The putative type I BVMOs sequences of interest were selected based on a minimum identity percentage of 35% and an E value close to 0. Finally, conserved domains and key sites were analyzed using the InterPro database (https://www.ebi.ac.uk/interpro/, 1 March 2024) and ClustalW alignments of these sequences to build the phylogenetic tree using the neighbor-joining method [37]. Around 1000 replicates were made using the MEGA-X software, Version 11.0.13 [38].

### 3.3. Cloning of MO-G35A

Considering that MO-G35A exhibited an 86% identity with the amino acid query sequence Sle_62070 and that typical type I BVMO domains were identified, the MO-G35A enzyme was selected for the study. For cloning, a six-histidine tag was added to the C-terminal of the protein sequence of MO-G35A. Then, the aminoacidic sequence was reverse translated with the preferred codons for *E. coli* using the SnapGene (Version 7.2.1) tool. Restriction sites for Ncol were added in the 5′ and stop codon plus restriction sites for Xhol were added into the 3′. The genes were synthesized and cloned into pET-22b and pET-28a by GeneUniversal (Newark, Delaware, USA) using the restriction enzymes Ncol and Xhol. In this way, the gene was cloned in frame with the peIB secretion signal into pET-22b for periplasmic production and cloned in pET28a for cytoplasmic production. The first-mentioned construction was transformed into *E. coli* NiCo21(DE3) and transformants were selected on LB agar plates containing 100 μg/mL ampicillin. Meanwhile, the latter construction was transformed into both *E. coli* NiCo21(DE3) and *E. coli* Shuffle T7 cells, and transformants were selected on LB agar plates containing 50 μg/mL kanamycin (Table 1).

### 3.4. Expression of MO-G35A

Pre-Culture 1 was produced by inoculating 10 mL of LB (Tryptone 10 g/L; yeast extract 5 g/L; NaCl 10 g/L) containing 100 μg/mL ampicillin (MO-G35A-p22N strain) or 50 μg/mL kanamycin (MO-G35A-p28N and MO-G35A-p28S strains), with a single transformant *E. coli* colony, and incubated at 230 rpm and 37 °C (*E. coli* NiCo21(DE3) cells) or 30 °C (*E. coli* Shuffle T7 cells) overnight. For Pre-Culture 2, 50 mL of LB with the corresponding antibiotics, were inoculated with 1 mL of Pre-Culture 1 and incubated at the corresponding temperature and shaken at 230 rpm for 4 h. Then, in a 2 L shake flask, 400 mL of TB (Tryptone 12 g/L; yeast extract 24 g/L; potassium hydrogen phosphate (Dibasic) 9.4 g/L; potassium phosphate (Monobasic) 2.2 g/L; glycerol 4 g/L) were inoculated with the appropriate volume of Pre-Culture 2, so it reached a starting OD600 of 0.06. Cultures were incubated under the same conditions described earlier, until they reached an OD600 of approximately 1. Then, an IPTG pulse was added for a final concentration of 0.5 mM. The same culture conditions were maintained for another 18 h.

Cells were harvested after centrifugation at 5000 rpm for 10 min at 4 °C. The supernatant was discarded, and the cell pellet was washed with 20 mM Tris-HCl. Centrifugation was repeated under the same conditions, and the pellet was collected and stored at −20 °C until further analysis.

### 3.5. Purification of Recombinant MO-G35A

The cell pellet from 50 mL of culture was resuspended in 25 mL of 50 mM Tris-HCl pH 7.5, 500 mM NaCl, 5 mM imidazole, and then sonicated during 2 min in 10/30 s On/Off cycles at a 40% amplitude. The resulting solution was centrifuged at 11,000 rpm and 4 °C for 30 min. The pellet was discarded while the supernatant was transferred to new tubes.

The supernatant was filtered using 0.2 μm filters and transferred to fresh sterile tubes. Chromatographic steps were carried out on FPLC system AKTA™ Avant 25 following the adapted methodology from Lehuedé et al. (2024) [61], where 20 mL of the samples were loaded in a Histrap^®^ FF 1 mL column, which was preequilibrated with binding buffer (50 mM Tris-HCl pH 7.5, 500 mM NaCl, 5 mM imidazole). Then, the column was washed with 20 mL of binding buffer. The elution was carried out in 8 mL of a linear gradient from 0 to 100% at a 0.5 mL/min flow of elution buffer (50 mM Tris-HCl pH 7.5, 500 mM NaCl, 500 mM imidazole) and collected in a 96 deep-well plate as 1 mL fractions and its absorbance at 280 nm was recorded. The fraction that showed absorbance at 280 nm was collected and stored at 4 °C for further analysis.

### 3.6. Enzyme Characterization

#### 3.6.1. MO-G35A Activity Assays

The enzyme was used directly from the soluble extract or purified (following the purification method described below), according to the experiment. For preparing the soluble extract, the frozen pellets were resuspended in the corresponding volume of 50 mM Tris-HCl pH 7.5, for starting with an OD600 equal to 40. The solution was then sonicated for 2 min in 10/30 s on/off cycles at a 40% amplitude, followed by centrifugation at 11,000 rpm and 4 °C during 30 min. The cell pellet was discarded while the supernatant was transferred to new tubes.

For evaluating MO-G35A catalytic activity, 50 µL of 200 mM Tris-HCl pH 7.5, 50 µL of 670 µM NADPH, 50 µL of 20 mM BHC and 50 µL of the enzyme (from the soluble extract or purified enzyme) were placed in a UV 96-well plate. Immediately after the enzyme was added, the plate was placed in a microplate reader (SPECTROstar Omega, BMG Labtech, Ortenberg, Germany), previously set at 30 °C, and absorbance was measured at 340 nm every 80 s, to determine NADPH disappearance. Two controls were included, where an equivalent volume of buffer replaced either the substrate or the enzyme.

When the purified enzyme was used, enzymatic units were obtained. One enzymatic unit (U) was defined as the µmol of NADPH consumed per minute by the enzyme, at a given pH and temperature conditions.

#### 3.6.2. Protein Quantification

Protein quantification was performed using the Bradford method, where 200 µL of Bradford reagent (Bio-rad) were mixed with 5 µL of the purified enzyme or soluble extract. After 10 min, the absorbance at 595 nm was measured using a microplate reader (SPECTROstar Omega). Then, protein concentration was determined through a BSA standard curve.

#### 3.6.3. MO-G35A Kinetic Parameters

To determine BHC kinetic parameters, enzyme activity was determined with 24.2 µg of purified enzyme by varying the concentration of BHC from 0 to 30 mM and the concentration of NADPH was fixed at 200 µM. For the determination of NADPH kinetic parameters, enzyme activity was determined with 13.7 µg of purified enzyme varying the concentration of NADPH from 0 to 250 µM and concentration of BHC was fixed at 5 mM. The activity assays were performed at 30 °C and pH 7.5. The kinetic parameters were calculated using the GraphPad Prism 8 program.

#### 3.6.4. pH Effect on MO-G35A Activity

To evaluate the enzyme optimal pH, 50 mM acetate buffer was used for pH from 3.7 to 5.6, 50 mM Tris-HCl for pH ranging from 5.8 to 8.0, 50 mM glycine-NaOH for pH between 8.6 and 10.6, and 50 mM sodium phosphate for pH from 11 to 11.9. Every enzymatic reaction was performed at 30 °C. Relative activity was calculated based on the pH where the enzyme had the highest activity.

#### 3.6.5. Temperature Effect on MO-G35A Activity

The effect of temperature on the enzyme activity was evaluated by carrying out activity assays as previously described at pH 7.5, but varying the temperature used, ranging from 18 °C to 57 °C, using a thermocycler (Bioer GeneExplorer^®^, Bioer, Hangzhou, China). Relative activity was calculated using the optimal temperature as 100%.

#### 3.6.6. MO-G35A Thermal Stability

The remaining enzyme activity was measured at pH 7.5 and 30 °C as previously described, after incubating MO-G35A during 5 and 10 min at a set temperature, ranging between 15 °C and 42 °C, in a thermocycler (Bioer GeneExplorer^®^).

#### 3.6.7. Solvent Effect on MO-G35A Activity

For evaluating the influence of certain solvents (acetone, acetonitrile, dichloromethane, DMSO, ethanol, methanol, isopropyl alcohol) on the catalytic capacity of MO-G35A, two activity assays were performed in a UV 96-well plate, as described earlier, but varying volumes of each solution added. For a solvent final concentration of 5%, 40 µL of 250 mM Tris-HCl pH 7.5, 50 µL of 20 mM BHC, 10 µL of 100% v/v solvent, 50 µL of 670 µM NADPH, and 50 µL of purified MO-G35A were added. NADPH disappearance was measured using a microplate reader (SPECTROstar Omega). Enzymatic reactions were performed at 30 °C.

For a solvent final concentration of 10%, the reaction mixture contained 30 µL of 334 mM Tris-HCl pH 7.5, 50 µL of 20 mM BHC 20 µL of 100% v/v solvent, 50 µL of 670 µM NADPH and 50 µL of purified MO-G35A.

#### 3.6.8. Ions Effect on MO-G35A Activity

To evaluate how the presence of different ions affects the enzyme catalytic capacity, an ionic compound (NaCl, MnCl_2_, CuSO_4_, KCl, CaCl_2_, or KI) was added to the enzymatic reaction mixture at 5 or 10 mM. A control was also performed by adding the same volume of MilliQ water. The activity assays were carried out as described earlier at pH 7.5 and 30 °C.

#### 3.6.9. Statistical Analysis

All experiments were performed in triplicate. The experimental data were analyzed using the GraphPad Prism 8^®^ software; analysis of variance (ANOVA) and Tukey tests with a 95% confidence level were carried out.

## 4. Conclusions

This investigation has successfully found, produced, and characterized the MO-G35A monooxygenase present in a *Streptomyces* strain isolated from the rhizosphere of *Lupinus oreophilus* in the Atacama Desert. Bioinformatic analysis revealed that MO-G35A is closely related to the previously described BVMO Sle_62070 from *Streptomyces leeuwenhoekii C34* and shares its activity towards BHC. The enzyme was successfully expressed in *E. coli*, with higher yields achieved using the pET-28a plasmid in the Shuffle T7 system, highlighting the importance of correct disulfide bond formation for its activity.

Characterization of MO-G35A showed optimal activity at 35–38 °C, retaining approximately 80% of its residual activity between 15 and 35 °C, and exhibiting kinetic parameters of *Km* = 0.06 mM and *kcat* = 0.15 s^−1^ for BHC. In particular, the enzyme demonstrated high stability in the presence of organic solvents and ions, making it suitable for applications in a wide range of industrial processes.

The present study contributed to the discovery of a novel enzyme from extremophiles, overcoming challenges related to enzyme stability under harsh conditions and expanding the repertoire of biotechnologically relevant enzymes. Overall, MO-G35A represents a promising candidate for further development of investigations towards exploring novel substrates and reactions and constructing new whole-cell biocatalysts. The coexpression of the dehydrogenase found downstream of the genomic sequence and the study of a suitable host for the redox cascade will allow the advancement towards its application in industrial processes.

## Figures and Tables

**Table 1:** Constructed strains expressing MO-G35A gene.

Strain	Plasmid	Host	Reference
MO-G35A-p22N	pET-22b	*E. coli* NiCo21(DE3)	This work
MO-G35A-p28N	pET-28a	*E. coli* NiCo21(DE3)	This work
MO-G35A-p28S	pET-28a	*E. coli* Shuffle T7	This work

## Data Availability

Data presented in this study are available from the corresponding author, upon reasonable request.
